# A Theory of Change for Guiding the Integration of Human-Centered Design Into Global Health Programming

**DOI:** 10.9745/GHSP-D-21-00334

**Published:** 2021-11-29

**Authors:** Anne LaFond, Montana Cherney

**Affiliations:** a John Snow Inc., Arlington, VA, USA.; b McKinsey & Company, Munich, Germany.

## Abstract

The goal of this theory of change is to provide funders, implementing partners, and designers a tool to increase the understanding and application of human-centered design (HCD) as an approach that enhances global health programming. The theory of change also aims to communicate the potential for HCD to introduce new and disruptive mindsets, practices, and techniques to global health programming and to clarify HCD’s potential value from the funding application process through to implementation. We seek to answer the key question: how do design and traditional global health practices and mindsets integrate to drive more people-centered, innovative solutions to health challenges and achieve common health sector and global health ecosystem goals?

## Abstract (precis)

How do design and global health practices and mindsets better integrate to drive more people-centered, innovative solutions to health challenges and achieve common health sector and global health ecosystem goals? This article discusses a theory of change for guiding the integration of human-centered design into global health programming.

## Key Messages

- Implementing partners, designers, and funders need tools and common language to increase the understanding and application of human-centered design (HCD) as an approach that enhances global health programming.
- To advance the integration of HCD and global health, designers and implementing partners should be able to articulate how HCD and global health practice can work together to support the achievement of health sector and global health ecosystem goals.

## INTRODUCTION

Human-centered design (HCD) is an approach to problem framing and solution generation that has gained wide acceptance and delivered measurable impact in the private sector,*^,^^1^^–^^3^ but has only recently been applied in global health.^4^^–^^8^ A body of experimentation and practice is emerging that delineates how HCD works on the ground, how HCD and health professionals are connecting their practices, and the benefits and challenges of integrating the 2 fields.^9^^–^^10^ Yet, the theoretical pathways of the influence of design in health programming remain poorly articulated.

This article draws on recent experience and early efforts to explain the influence of HCD in health programming. In 2019, Design for Health^11^ convened a group of global health and design practitioners to draft a theory of change (TOC) to convey the role of HCD in global health programming and its proposed contribution to realizing global health goals. Building on these early initiatives, we propose a second-generation TOC on the **integration** of HCD and global health programming for reflection and testing. This TOC intends to increase understanding of HCD and its application as an approach that brings new, often disruptive framing, mindsets, and practices in the sense that it challenges traditional ways of thinking and working, enhances program processes and effects, and promotes agency and self-determination in problem solving. The draft TOC also provides funders, implementing partners, and designers with a tool for communicating the influence of design in global health practices, and clarifying HCD’s potential value, from human-centered problem framing, intervention design, and collaboration to implementation and evaluation of program impact. The TOC aims to answer how do design and traditional global health practices and mindsets better integrate to drive more people-centered, innovative solutions to health challenges and achieve common health sector and global health ecosystem goals?

The theory of change aims to answer how do design and traditional global health practices and mindsets better integrate to drive more people-centered, innovative solutions to health challenges?

## RATIONALE FOR INTEGRATING HCD IN GLOBAL HEALTH

HCD in the health sector is defined as an iterative and participatory approach to building global health interventions that achieve health impact.^11^ By placing people at the center during product, service, and program development and continuously testing and iterating solutions throughout the design and implementation process, HCD ensures that people’s needs, desires, and contexts inform key decisions and that solutions are relevant, compelling, and tailored to specific contexts. Design is a craft and a discipline that applies a distinct mindset and skill set to a creative problem-solving process.^11^ It can be applied differently, in terms of extent and intensity, depending on the challenges, timelines, and resources available, and therefore can serve numerous purposes. Design methods can be applied to help spark new ideas, thinking, and concepts; to deliver specific outputs as part of a larger program; as an “ingredient” in combination with other approaches across the program cycle; and as an end-to-end process, with the program scoped to match the design process, from informing program design to implementing solutions.^11^

We posit that more seamlessly integrating HCD skill sets and mindsets into global health programming has the potential to strengthen global health actors’ capabilities and practices and enable them to adopt new, often catalytic approaches and solutions. By activating 3 core tenets of design—multidisciplinary collaboration, centering on people in their contexts, and creativity and iteration—HCD contributes to the realization of health sector investment goals: improved population health and improved health systems performance.^11^^–^^14^ In addition, we propose that integration of HCD also has the potential to enable the global health ecosystem to work in ways that enhance equity and inclusion and create greater openness to innovation and new ways of programming.^5^^,^^15^ Based on the first TOC iteration and recent experience applying HCD in global health, we describe in the next-generation TOC 2 theoretical pathways of change associated with the integration of HCD in global health programming.

HCD contributes to the realization of health sector investment goals: improved population health; improved health systems performance; and increased equity, inclusion, and innovation in the global health ecosystem.

## THE THEORY OF CHANGE

This (draft) TOC illustrates the influence of HCD in global health in 2 related domains: the health sector and the global health ecosystem. It outlines the pathways to achieving health sector and global health ecosystem goals that emanate from the influence of HCD in global health programming. The TOC articulates how HCD can strengthen existing processes and introduce new approaches to problem framing and the generation and implementation of solutions, working in concert with stakeholders such as health service clients, providers, managers, and funders to improve health as well as the institutions that frame, direct, and manage investment and priorities in health sectors in low resource settings. As the field has not yet generated a large body of evidence to inform the logic of these pathways, we have drawn on collective experience and expert consultation in developing the TOC. We hope that articulating these pathways invites testing and interrogation to advance understanding of this topic, offers teams the opportunity to continue to apply and evolve a common framework using a shared language, and increases and strengthens the evidence.

### Framing the 2 Pathways of Change: Goals, Preconditions, and Outcomes of Integrating HCD

At the core of the TOC are universally accepted health sector goals shared among global health and HCD practitioners: improved health system performance and improved population health.^16^^–^^19^ The value of integrating HCD must be assessed against its contribution to achieving these fundamental aims of health investment in low-resource settings. The TOC asserts that when HCD is integrated into global health programming, it can improve the likelihood of realizing these goals. In the second pathway, the TOC further posits at the goal level that HCD not only helps to strengthen systems and improve health, it also influences the global health ecosystem that encompasses the health sector by integrating program processes and products that enhance equity and inclusion, thereby strengthening traditional ways of working. For example, HCD helps to build capacity among individuals and within communities to play a more active role in their care experiences and stimulates ecosystem openness to innovation, collaboration, and human-centered programming and investment practices. These 2 theoretical pathways of change intersect and may occur simultaneously but advance at different rates of change. As experience integrating HCD in the health sector grows it builds momentum for change in the global health ecosystem.^20^^–^^24^

Moving from the bottom to the top of the TOC (Figure), the TOC consists of related layers of cause and effect, beginning with HCD’s influence on global health programming processes and intervention shaping, which is represented as **HCD inputs** to the programming process and the **outputs and outcomes of practicing HCD.** In the subsequent upper layers, the TOC illustrates how HCD operates along 2 pathways (the health sector and the global health ecosystem) to help create some of the necessary conditions for realizing health sector and ecosystem goals, which are represented as the **outcomes of integrating HCD** and **preconditions.** There are, of course, many other preconditions for achieving these goals, but for this discussion, we focus on preconditions linked to the integration of HCD in global health programming. The 2 pathways of influence of HCD on global health are related and mutually reinforcing. We discuss each pathway separately below, describe how HCD works in practice, and how it can drive impact and change.

**FIGURE:**
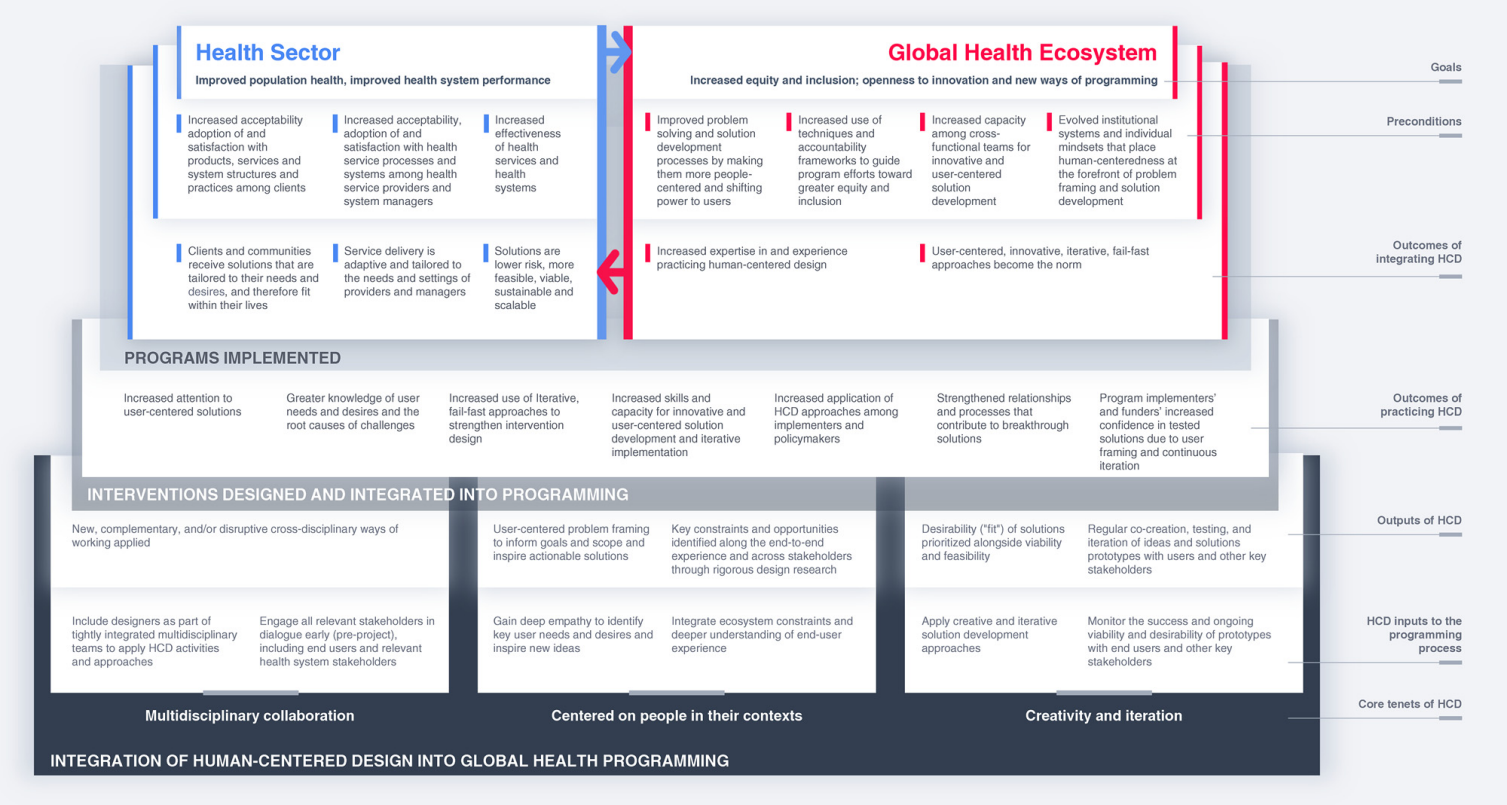
A Theory of Change for Guiding the Integration of Human-Centered Design Into Global Health Programming

As stated earlier, the design process can be applied differently depending on the challenges, timelines, and resources available, and therefore can serve numerous purposes. HCD enhances traditional global health programming approaches and introduces new ways of thinking and working as a consequence of its 3 core tenets^11^^–^^14^:

- Engagement of multidisciplinary teams (including end users and health system stakeholders) in problem framing and the co-creation of solutions
- Centering of users’ needs, desires, and contexts and identification of ecosystem constraints
- Use of cycles of creative and iterative solution development and the testing and monitoring of the desirability and viability of solutions for end users and key stakeholders

HCD enhances traditional global health programming approaches and introduces new ways of thinking and working.

## HCD IN PRACTICE: ENHANCING INTERVENTION DESIGN AND IMPLEMENTATION

### Inputs and Outcomes of Practicing HCD

In practice, HCD is typically introduced at an early stage of problem framing and exploration, guiding a process of user-centered solution development and testing that generates 1 or more products, services, or interventions as components of the overall program.

Many HCD activities and approaches (**HCD inputs** at the bottom of the Figure), such as strengthening cross-disciplinary collaboration and user involvement in intervention design, complement existing health program planning practices. Others, such as introducing phases of early-stage user insight generation and testing and adapting solutions before full-scale implementation, often challenge accepted practices such as rigid adherence to program designs agreed on at the point of initial funding. To illustrate how HCD influences global health programming, the Figure groups the inputs according to the 3 core tenets of HCD: multidisciplinary collaboration, centering on people in their contexts, and creativity and iteration.^11^^–^^14^

The core HCD tenet of **multidisciplinary collaboration** posits that the practices of creating tightly integrated multidisciplinary teams that apply HCD activities and approaches and engaging all relevant stakeholders early introduces new collaborative and cross-disciplinary ways of working. In fact, incorporating a broader set of stakeholders in the conversation from the start can help teams to identify and overlay the needs and desires of a broader group of stakeholders and enhance community engagement.^25^^–^^26^ It also helps to ensure that discomfort or concerns between stakeholders that might arise in solution development are acknowledged and addressed early.

The second tenet, framing problems and generating solutions **centered on people in their contexts** is at the heart of HCD. While global health practitioners have taken a people-centered approach for decades, design borrows from approaches like ethnography and psychology and brings new dimensions that complement and enhance traditional approaches and particular attention to individual identities and lived experience that is often missing in global health programming.^27^^–^^28^ Using desirability and fit as their compass, designers invite end users to describe not just what they need, but what they *want,* and how what they want can best fit into their lives.

Global health practitioners have taken a people-centered approach for decades, but design complements and enhances traditional approaches and brings attention to individual identities and lived experience that is often missing in global health programming.

At the outset of a project that integrates HCD, the team often conducts design research to gain a deeper understanding of and empathy with users in their contexts; identify their needs, desires, and contexts; and inspire new ideas for solving a health challenge or achieving a health goal by looking at their experience in an integrated manner rather than assuming that health decisions are made in isolation of other factors.^29^ Another way that design ushers in new layers of understanding to the problem-framing process is by using creative storytelling tools to illustrate constraints and opportunities along the end-to-end user experience and across the broad ecosystem of stakeholders. These tools, including personas, journey maps, and ecosystem maps, offer teams more relatable, often visual, ways to help align stakeholders by employing new ways of becoming aware of users’ experiences and the interconnections between stakeholders.

**Creativity and iteration,** the third core tenet of HCD, borrows largely from tools and approaches widely employed in the private sector in which users and other stakeholders collaborate on the development of solutions and engage in divergent and convergent thinking processes.^30^ Creativity is at the heart of HCD’s open-minded exploration and generation of ideas to deepen understanding of problems and their root causes, followed by facilitated discussions and processes like prototyping (i.e., iterative, fail-fast testing, and adapting) to optimize the selection of solutions.^31^ Ongoing involvement of users—from brainstorming to codesigning and prototyping—helps to ensure that the desirability and fit of solutions are prioritized alongside viability and feasibility.^32^^–^^33^

The **outcomes of practicing HCD** take the form of greater knowledge and prioritization of users’ needs and contexts, strengthened relationships and multidisciplinary partnerships in health sector programming, and increased use of iterative, prototyping cycles. Designing not just for but *with* users and monitoring the success, viability, and desirability of prototypes with end users and other stakeholders throughout solution development help to increase implementers’ and funders’ confidence in solutions. Additional outcomes of program planners’ and implementers’ ongoing engagement in HCD include the evolution of human-centered mindsets over time in these actors, an increased capacity for innovative and iterative solution development and implementation, and a greater willingness to apply HCD along with traditional health programming practices.

### The Outcomes of Integrating HCD: 2 Pathways

HCD inputs come together during project planning to produce products, interventions, services, and strategies that have been tested with users in their context and that can become small or large components of overall health sector investments whether HCD is used as a spark, ingredient, or end-to-end.^11^ Integrating HCD also introduces program managers and participants to new skills, practices, and mindsets that prioritize human-centered programming and creative problem solving. Over time, as HCD processes are repeatedly applied, the new skills and practices can reorient program planners’ approaches to intervention design and implementation and increase inidividuals’ and communities’ sense of agency.^8^^,^^34^

The upper 3 layers of the Figure (**goals, preconditions, and outcomes of integrating HCD**) delineate the outcomes of implementing HCD-influenced programs and integrating HCD-influenced processes into global health practice. The health sector pathway on the left side focuses on the key outcomes of tailoring and de-risking interventions, while the global health ecosystem pathway on the right emphasizes orienting investment toward users and user agency and innovation.

## HEALTH SECTOR PATHWAY

When HCD is integrated into global health programming, clients and communities receive solutions tailored to their needs and health service and behavior change interventions are carefully aligned with providers’ and managers’ needs and settings. The design process helps teams to shape and implement interventions that better meet the needs and desires of users and stakeholders across the health system, reducing risk and optimizing the likelihood that the desired behaviors, products, and services will be adopted and accepted.^11^^,^^35^ As such, HCD helps to create the following important **preconditions** for achieving health sector goals:

1. Increased **acceptability and adoption of and satisfaction with** global health products, services, system structures, and practices **among clients**
2. Increased **acceptability and adoption of and satisfaction with** health service processes and systems **among health service providers and system managers** (i.e., key system actors)
3. Increased **effectiveness of health services and health systems** (i.e., solutions that work for people in their context and meet standards for accessibility, availability, quality, affordability, and desirability)

The design process helps shape and implement interventions that better meet the needs and desires of users and stakeholders across the health system, optimizing the likelihood that the desired behaviors, products, and services will be adopted and accepted.

### Preconditions: Acceptability, Adoption, and Satisfaction

One of the key benefits of integrating HCD is that design processes can help to shape a product or service to increase its desirability in the eyes of the client and drive acceptance, adoption, and satisfaction—critical elements in improving health and health systems performance. HCD can also help tailor services, delivery settings, and processes so they are more acceptable to service managers and providers, which increases the likelihood of adoption and continued use of the service delivery practices. In both cases, the user (i.e., the person who seeks better health or the actor in the system who contributes to better health) is paramount in the design process. In fact, in **the early stages of HCD-led intervention/project design**, the desirability of the product, intervention, or service takes precedence over other considerations like cost and efficiency to drive **acceptability and adoption.** The user’s perspective continues to be a critical success factor throughout solution development and implementation, with desirability one of the key metrics that guide design decisions.^36^ Designers posit that if people are not motivated to use a service or do not feel successful, rewarded, or delighted when they practice a health-promoting act or service, they are not likely to become a steadfast “customer” of the service or doer of the behavior.

By exploring how those involved in a health service experience it from end to end of the journey, HCD also helps to reveal ways to improve health service delivery. Design processes manage the conflicting user concerns that often arise and refine possible solutions through iterative prototyping cycles with users. HCD tailors solutions to users’ contexts, needs, and aspirations by collaborating closely with end users—clients, community members, health care workers, and other health system stakeholders—to enhance “fit” and acceptability. In this manner, HCD seeks to disrupt traditional program planning by prioritizing users’ needs and desires early in problem framing and later, before widespread implementation, by testing the feasibility of introducing and sustaining the solution in the community or health system context.

### Precondition: Effectiveness of Solutions

Beyond improved product or service uptake, applying HCD approaches can enhance the overall intervention strategy used to define and deliver a product or service or introduce health-seeking or health-positive behaviors. By designing and testing for feasibility and viability, the other key metrics that guide design decisions, HCD processes reduce the risk of failure of an intervention as a whole thus enhancing the likelihood of success. A design-influenced approach embodying substantial user engagement in problem framing, solution generation, and iterative testing and adapting increases the likelihood that program managers will identify implementation challenges and needs early on.^37^ It provides them with a more nuanced understanding of the feasibility of implementation in a specific context, helping to avoid costly, late-stage learning about the fit between the intervention and the health system or community context. For example, prototyping delivery strategies, partnerships, and cost-recovery mechanisms in different settings with different population segments increases confidence in the design of an intervention, its implementation trajectory, and its potential for success.

## GLOBAL HEALTH ECOSYSTEM PATHWAY

Introducing and enhancing HCD’s people-centered, collaborative practices provides opportunities to enhance global health ecosystem structures and practices. This includes advancing actors’ ability and willingness to apply creativity and innovation in developing health interventions and to focus on increasing equity and inclusion in the health sector.^5^ With respect to innovation, the global health field is somewhat resistant to creative problem-solving approaches, including the strategy of “failing fast” to find solutions that fit.

HCD enables global health actors to experiment with new ways of working that incorporate a learning and testing framework, which can disrupt or enhance existing practices.^25^ Conversely, HCD can also benefit from decades of global health experience and rigor assessing feasibility requirements and evaluating program effectiveness—experiences that are needed to ensure that HCD-influenced solutions are adapted for system sustainability and scale-up. The fields of global health and HCD also share a commitment to equity and inclusion that make them good allies, and HCD introduces additional techniques for integrating inclusive practices during intervention design and implementation.

As HCD is integrated more systematically in health programming, it is beginning to blend with traditional processes to create critical preconditions for system-level adoption of new approaches to intervention design and collaboration. HCD also helps to position the ecosystem to achieve greater equity and inclusion in its practices and its outcomes.^5^ At the ecosystem level, HCD contributes to the following **preconditions** for greater equity, inclusion, and innovation:

- Improved problem-solving and solution development processes as a result of making the processes more people-centered and shifting power to users
- Increased use of accountability techniques and frameworks to guide program efforts toward greater equity and inclusion
- Increased capacity of cross-functional teams to employ innovative, user-centered solution development, including openness to adapting solutions and systems to sustain or improve impact
- Evolved institutional systems and mindsets of funders, implementers, and other health system stakeholders that place human centeredness at the forefront of problem framing and solution development

## CONCLUSION

This TOC illustrates pathways to achieving key health sector and global health ecosystem goals that emanate from the influence of HCD on health programming processes, such as problem framing, defining solutions and interventions, and implementing solutions that are tailored to users’ needs, desires, and contexts. The TOC also depicts the role of HCD in reorienting the mindsets and actions of donors, program developers, health program managers, and service providers toward creative, people-centered, iterative approaches grounded in learning.

Typically, TOCs focus on a specific intervention or program. This TOC seeks to articulate and evolve an understanding of broader global health programming practices. We hope to engage a community of funders, implementing partners, and designers in a dialogue about the integration of HCD and global health. Most importantly, we aspire to create a common framework and language to answer the question: how do design and traditional global health practices and mindsets integrate to drive more people-centered, innovative solutions to health challenges and achieve common health sector and global health ecosystem goals?

We encourage funders, global health practitioners, designers, and evaluators to use this TOC to:

- Introduce and explain key HCD processes in the context of global health programming and articulate its influence pathways
- Increase understanding of and enhance alignment on how HCD can complement traditional approaches to achieving health sector and global health ecosystem goals, thereby optimizing collaboration between designers and global health practitioners
- Develop framing and metrics to track the integration of HCD into global health programming
- Test the pathways of change and generate evidence on the process and outcomes of integrating HCD into global health programming

## Notes

**Peer Reviewed**
